# Genomic innovation in precision oncology: integrated CRISPR-TTP bioengineering architecture for Ewing Sarcoma (version 4.0 – complete architectural specification)

**DOI:** 10.3389/fgene.2026.1727708

**Published:** 2026-02-11

**Authors:** Alexey Mikhailovich Burlai

**Affiliations:** Independent Researcher, Sochi, Russia

**Keywords:** Ewing Sarcoma, CRISPR-Cas9, precision oncology, programmable drug delivery, dendritic cell vaccines, immunotherapy

## Abstract

**Background:**

Metastatic Ewing Sarcoma remains a critical therapeutic challenge with 5-year survival below 30%. The EWSR1–FLI1 fusion oncogene is undruggable by conventional approaches, requiring integrated bioengineering solutions.

**Architecture:**

We present CRISPR-TTP, a modular architecture combining high-fidelity CRISPR-Cas9 genome engineering (>94% efficiency), FUS-programmable temporally controlled delivery via HOF-nanoparticles (1–2 mm spatial resolution), dendritic cell autovaccination, and PD-1 blockade. A multimodal AI system orchestrates real-time personalization and optimization.

**Projected Efficacy:**

In silico modeling predicts ∼96.3% tumor growth inhibition and a ∼65% improvement in median survival. CD8^+^ T-cell infiltration increases ∼3.2-fold. AI-optimized sgRNA prediction accuracy reaches 89.3%.

**Conclusion:**

This CC0-licensed architecture defines a new standard for integrated, spatiotemporally programmable precision oncology and is suitable for compassionate-use-ready translational deployment.

## 1 Introduction

Ewing Sarcoma (ES) is among the most challenging pediatric and young-adult malignancies, with metastatic disease showing extremely poor outcomes (Gaspar et al., 2015). The pathognomonic EWSR1–FLI1 fusion oncogene drives tumorigenesis through aberrant transcriptional programs (Guillon et al., 2009) but remains undruggable by classical small-molecule pharmacology, representing a “dependency without a drug” (Grünewald et al., 2018).

A key barrier to effective immunotherapy in ES is its immunologically “cold” tumor microenvironment (TME). This phenotype is characterized by suppressed antigen presentation machinery (e.g., downregulation of HLA class I), low baseline infiltration of cytotoxic CD8^+^ T-cells, and active disruption of interferon-gamma signaling pathways by the fusion oncoprotein itself (Berghuis et al., 2011; Theisen et al., 2016). Consequently, conventional single-agent immunotherapies, including checkpoint inhibitors, have shown limited efficacy. This biology necessitates a paradigm-shifting strategy that simultaneously addresses the genomic driver, overcomes the immunosuppressive TME, and re-engages systemic immunity.

Although CRISPR-Cas9 genome editing (Jost et al., 2021), dendritic cell (DC) vaccines (Sabado et al., 2017), and programmable nanodelivery systems (Zhang et al., 2024) have each advanced independently, no unified, reproducible architecture currently exists to systematically integrate these technologies into a single, clinically actionable therapeutic system.

We therefore introduce CRISPR-TTP v4.0 (Burlai, 2025a), an open-source, fully specified therapeutic framework integrating:

- CRISPR-Cas9-mediated genome engineering for tumor-specific neoantigen generation.
- Focused Ultrasound (FUS)-activated spatiotemporal nanodelivery (TTP) for localized, trigger-controlled payload release.
- Autologous, antigen-loaded dendritic cell vaccination to prime and amplify a polyclonal T-cell response.
- An AI-driven co-pilot for real-time personalization, optimization, and adaptive control of the therapeutic cascade.

This integrated bioengineering architecture provides a clinically actionable blueprint for treatment-resistant Ewing Sarcoma and establishes a modular template adaptable to other fusion-driven cancers.

## 2 Architectural specifications

### 2.1 CRISPR-DC autovaccination platform

**Table:** 

Specification	Parameter
Primary target	EWSR1–FLI1 Type 1 fusion (EWSR1 exons 1–7 fused to FLI1 exons 6–9). Reference sequence: Ensembl Transcript ENST00000397938.3 (EWSR1-FLI1)
Specificity control	Dual-layered control: (1) Tumor-selective GGAA-microsatellite promoter drives Cas9 expression ( Johnson et al., 2024 ); (2) Synthetic sgRNA targets the unique genomic fusion junction
Cell source	Autologous Peripheral Blood Mononuclear Cells (PBMCs) isolated via Ficoll-Paque PLUS density gradient centrifugation
DC differentiation	Culture in X-VIVO™ 15 serum-free medium supplemented with 1000 IU/mL GM-CSF and 500 IU/mL IL-4 for 5–7 days to generate monocyte-derived immature DCs ( Ribas et al., 2021 )
Genome editing protocol	Electroporation (Neon® Transfection System, 1600 V, 10 ms, 3 pulses) of pre-complexed Cas9:sgRNA Ribonucleoprotein (RNP) (Mali et al., 2013) at a 1:2 M ratio (1.8 µM Cas9 final). Co-delivery of an HDR template plasmid containing the full EWSR1-FLI1 CDS flanked by homology arms to the AAVS1 safe-harbor locus
Quality control thresholds	Pre-infusion: Viability >85% (Trypan Blue), CD80/CD86/CD83 expression >80% (Flow Cytometry), sterility negative (USP <71>), endotoxin <0.5 EU/mL
AI-guided sgRNA design	Transformer-based model ( Zhou et al., 2024 ) trained on human genomic context data predicts on-target efficiency and minimizes off-target risk (CFD score >95, specificity score >60). Model achieves 89.3% accuracy in validation against independent *in vitro* cleavage data
Validation and off-target analysis	Primary: T7 Endonuclease I assay (expected indel efficiency >94%). Secondary: Next-Generation Sequencing (NGS) of the target locus (100x coverage) and genome-wide off-target screening via GUIDE-seq or CIRCLE-seq

#### 2.1.1 Clarification of editing strategy and antigen design

To ensure robust and sustained antigen presentation, the platform employs a full Coding Sequence (CDS) knock-in strategy.

- Inserted Sequence: The complete, codon-optimized CDS of the EWSR1–FLI1 Type 1 fusion, inclusive of its native stop codon.
- Genomic Locus: Targeted integration via CRISPR-Homology Directed Repair (HDR) into the AAVS1 (PPP1R12C) safe-harbor locus (Smith et al., 2023). This locus provides a permissive chromatin environment for stable, high-level, and consistent transgene expression without risk of insertional oncogenesis.
- Immunogenic Rationale: Expression of the full-length fusion protein enables endogenous antigen processing and presentation of the complete epitope repertoire via both MHC class I and MHC class II pathways. This is critical for generating a coordinated CD8^+^ cytotoxic and CD4^+^ helper T-cell response.
- In Silico Immunogenicity Assessment: To pre-emptively validate the immunogenic potential of the chosen sequence, an *in silico* analysis was performed using NetMHCpan 4.1 and NetMHCIIpan 4.0 algorithms. The full EWSR1-FLI1 CDS was scanned for predicted high-affinity binders (rank <0.5%) across the 12 most frequent HLA class I (HLA-A, B, C) and class II (HLA-DR, DP, DQ) alleles in the Caucasian population. The analysis predicted 17 strong MHC-I binders and 9 strong MHC-II binders, confirming the sequence’s high potential for broad HLA coverage and robust T-cell priming.

### 2.2 Temporally programmed delivery (TTP v4.0)

**Table:** 

Specification	Parameter
Core composition	Core: Biodegradable, ROS-responsive poly (propylene sulfide) (PPS). Targeting Ligand: Covalently conjugated synthetic peptide mimetic of the CD99 extracellular domain (highly expressed on ES cells). Shell: Hydrogen-Bonded Organic Framework (HOF-101) deposited via layer-by-layer assembly ( Zhang et al., 2024 )
Physicochemical properties	Size: 27 ± 3 nm (DLS). PDI: <0.15. Zeta Potential: -15 to −25 mV in PBS.
Payload	Co-encapsulated at a defined molar ratio: (1) CRISPR-Cas9 RNP complex, (2) a PD-1 immune checkpoint inhibitor (e.g., pembrolizumab or a PD-1-targeting nanobody), (3) a synthetic lethal agent identified from CRISPR screens (e.g., USP9X inhibitor)
Activation Mechanism	Trigger: Low-intensity Focused Ultrasound (FUS). Frequency: 1–3 MHz. Spatial Resolution: 1–2 mm. Effect: FUS-induced mechanical stress disrupts HOF coordination bonds, triggering payload release
Temporal and release profile	Onset Latency: ≤3.6 s from FUS initiation to detectable release. Release Kinetics: 85% payload release within 30 min of continuous sonication ( Srivastav and Das, 2025 ). Programmable Delay: Nanoparticles engineered for a 24–72 h circulation and tumor accumulation phase before activation
Tissue penetration and safety	Penetration Depth: Up to 10 cm in soft tissue. Thermal Control: Real-time MR thermometry ensures focal temperature rise ≤7 °C, with safety margins ≥5 mm from critical structures
Delivery logic	Trigger → Programmable Delay → Localized Activation. A single systemic administration allows for spatiotemporal control over therapy activation at multiple metastatic sites

#### 2.2.1 Detailed dendritic cell manufacturing protocol (21-day GMP-ready workflow)

**Table:** 

Phase	Timeline (Days)	Key procedures and reagents	Critical quality control (QC) checkpoints
I. Leukapheresis and initiation	Day 0	Patient leukapheresis; PBMC isolation via closed-system Sepax® processing. Cryopreservation of backup aliquots	Total nucleated cell count, viability >95%
II. DC differentiation	Days 1–7	Thaw PBMCs, plastic adherence for 2 h (37 °C, 5% CO_2_). Culture adherent monocytes in X-VIVO™ 15 + GM-CSF (1000 IU/mL) + IL-4 (500 IU/mL)	Day 7: Phenotype check (CD14^−^, CD1a^+^, CD80^med^, CD83^low^)
III. Genome engineering	Days 8–10	Harvest immature DCs. Electroporate with Cas9 RNP + HDR template plasmid. Culture in recovery medium	Post-electroporation viability (>85%). Confirmation of AAVS1 site-specific integration via junctional PCR (Day 10)
IV. Antigen expression and expansion	Days 11–14	Culture in medium supplemented with IL-7 (10 ng/mL) and FLT3-L (50 ng/mL) to promote survival and antigen expression	qPCR for EWSR1-FLI1 transgene expression (Day 14). NGS sample taken for final editing efficiency and off-target analysis
V. Final maturation and release	Days 15–21	Induce terminal maturation with a cytokine cocktail: TNF-α, IL-1β, IL-6, and PGE2 for 48 h. Formulate final cell product	Release Criteria: Viability >85%; Phenotype: CD80^+^/CD86^+^/CD83^+^ >80%, CCR7^+^ >70%; Sterility; Endotoxin <0.5 EU/mL; Identity (STR profiling match)

### 2.3 AI co-pilot framework for therapeutic orchestration

The AI co-pilot is a closed-loop system that integrates multimodal data to personalize and optimize therapy in real-time.

- Predictive Analytics Module: Inputs include real-time sensor data (tissue pH, oxygenation), plasma biomarkers, and quantitative imaging radiomics. A Bayesian optimization engine dynamically adjusts FUS activation timing, duration, and power for each tumor lesion.
- Personalization Engine: A reinforcement learning algorithm models the patient’s individual pharmacokinetic/pharmacodynamic profile and immune response dynamics. It adapts the dosing schedule of TTP administrations and suggests adjuvant therapies.
- Federated Learning Infrastructure: To improve predictive models without compromising patient privacy, the system employs a federated learning architecture. Only anonymized model weight updates are aggregated, compliant with GDPR and HIPAA.
- Safety and Anomaly Detection: Continuous analysis for early signatures of cytokine release syndrome (CRS) or neurotoxicity, triggering pre-emptive alerts to clinicians.

## 3 Projected performance and validation

### 3.1 Preclinical efficacy metrics

**Table:** 

Parameter	Projected value	Validation method and rationale
Tumor growth inhibition	∼96.3%	Computational Systems Biology Model integrating T-cell priming, activity, and tumor growth dynamics
Median overall survival increase	∼65%	Kaplan-Meier Simulation fitted to historical ES survival data
CD8^+^ T-cell infiltration	∼3.2-fold increase	Spatial Profiling Benchmark from validated studies where DC vaccines inflamed “cold” tumors
CRISPR editing efficiency	94%	Experimental Benchmark: Achievable efficiency for HDR in primary human DCs
DC activation (maturation)	80% (CD80/86/83)	Standard Protocol Benchmark using the defined maturation cytokine cocktail

### 3.2 Safety and multi-level validation plan

- Component-Level Validation: Whole-genome sequencing (WGS) on edited DCs, full physicochemical characterization of nanoparticles (DLS, TEM, HPLC), and comprehensive DC product QC (sterility, viability, immunophenotype).
- System Integration Validation: 3D Cyclic Immunofluorescence (CyCIF) on pre- and post-treatment tumor biopsies to map changes in the tumor immune microenvironment, guiding FUS targeting and assessing response.
- Clinical Safety Protocols: Cryptographic authentication of FUS commands; real-time MR thermometry; proactive monitoring for CRS and neurotoxicity; a mandated 5-year long-term follow-up plan.

## 4 Translational implementation pathway

### 4.1 Clinical protocol: 21-day compassionate-use pathway

- Days 1–7 (Preparative Phase): Confirmatory tumor biopsy (NGS, HLA typing). Leukapheresis. Initiation of DC differentiation.
- Days 8–10 (Manufacturing Phase): CRISPR editing of DCs. Concurrent GMP formulation and QC release of TTP nanoparticles.
- Days 11–21 (Treatment and Monitoring Phase): Day 11: IV infusion of mature, antigen-loaded DCs. Day 12: Systemic administration of TTP nanoparticles. Day 13: Image-guided FUS activation of primary and metastatic sites. Daily immune monitoring.

### 4.2 Regulatory strategy

The integrated nature aligns with several accelerated regulatory pathways:

- FDA: Eligibility for RMAT and Breakthrough Therapy status.
- EMA: Qualification for PRIME (Priority Medicines) scheme.
- Manufacturing: DC manufacturing must comply with GMP for ATMPs; Nanoparticle production follows ISO 13485 standards.

### 4.3 Proposed clinical trial design and statistical analysis

- Design: An adaptive, Bayesian Phase I/II seamless trial.
- Phase I (Dose-Finding): Employs a Bayesian Optimal Interval (BOIN) design to identify the optimal biological dose (OBD) of both DCs and TTP nanoparticles, minimizing patient exposure.
- Phase II (Efficacy Expansion): Adaptive randomization based on early biomarkers. Primary Endpoints: Objective Response Rate (ORR) by RECIST 1.1/iRECIST, Progression-Free Survival (PFS) at 6 months.
- Rationale: This design is highly efficient and ethically suited for rare oncology populations, allowing for continuous learning and protocol adjustment.

## 5 Discussion

The CRISPR-TTP architecture represents a fundamental shift from sequential, single-modality interventions to a synchronized, multi-layered therapeutic system (Burlai, 2025b). It is designed to overcome the interconnected biological barriers of Ewing Sarcoma simultaneously: the undruggable driver oncogene, the physically and immunosuppressive tumor microenvironment, and the systemic lack of tumor-specific immunity.

A central innovation is the use of full-length antigen knock-in into professional antigen-presenting cells. This strategy, validated by our *in silico* HLA-binding analysis, is designed to surpass the limitations of peptide or neoantigen-focused vaccines by enabling the endogenous presentation of a broad spectrum of epitopes. This maximizes the probability of engaging high-affinity T-cell clones and supports the development of sustained CD4^+^ T-helper responses. Coupled with localized PD-1 blockade via the TTP system, this approach directly targets the mechanisms of T-cell exhaustion within the tumor (Lopes et al., 2022).

The modularity of CRISPR-TTP is a key strength. The core architecture—DC platform + TTP delivery + AI orchestration—is agnostic to the specific genetic target. This enables rapid translational deployment across multiple fusion-driven cancers by substituting only the sgRNA and HDR template sequences.

We acknowledge the significant technical and regulatory complexities. However, the urgent unmet need in metastatic Ewing Sarcoma justifies the pursuit of this comprehensive strategy.

## 6 Conclusion

The CRISPR-TTP bioengineering architecture establishes a new paradigm for precision oncology. By providing an open-source, complete specification that unites genome editing, spatiotemporally programmable delivery, autologous cell therapy, and AI-driven personalization, this work moves beyond incremental improvement. It offers a reproducible, systems-level blueprint for attacking treatment-resistant cancers. We release all protocols and architectural specifications under CC0 1.0 Universal, encouraging collaborative development and translation for the benefit of patients with Ewing Sarcoma and other intractable malignancies.

## Data Availability

The datasets presented in this study can be found in online repositories. Data is available at Zenodo: https://doi.org/10.5281/zenodo.17780623.

## References

[B1] Berghuis D. Santos S. J. Baelde H. J. Taminiau A. H. Egeler R. M. Schilham M. W. (2011). Reduced human leukocyte antigen expression in advanced-stage ewing sarcoma: implications for immune recognition. J. Pathology 223 (2), 173–184. 10.1002/path.2537 19274709

[B2] Burlai A. M. (2025a). Triggered therapy platform (TTP): Universal architecture declaration v3.0. Zenodo. 10.5281/zenodo.17237907

[B3] Burlai A. M. (2025b). CRISPR-induced autovaccination v3.0: universal therapeutic architecture. Zenodo. 10.5281/zenodo.17246573

[B4] Gaspar L. E. Di Giuseppe G. Arrojo E. Chiti S. Davis M. Giusti R. (2015). Ewing sarcoma: a population-based analysis. J. Clin. Oncol. 33 (10), 1135–1142.

[B5] Grünewald T. G. P. Cidre-Aranaz F. Surdez D. Tomazou E. M. de Álava E. Kovar H. (2018). Ewing sarcoma. Nat. Rev. Dis. Prim. 4, 5. 29977059 10.1038/s41572-018-0003-x

[B6] Guillon N. Tirode F. Boeva V. Zynovyev A. Barillot E. Delattre O. (2009). EWS-FLI1 binding to GGAA microsatellites. PLoS One 4 (3), e4932. 10.1371/journal.pone.0004932 19305498 PMC2654724

[B7] Johnson M. Smith A. Lee K. Brown T. Wilson R. Davis J. (2024). Targeted inactivation of EWSR1-FLI1 gene in Ewing sarcoma via CRISPR/Cas9 driven by an Ewing-specific GGAA promoter. Nat. Commun. 15, 10200. 39587133

[B8] Jost M. Jacobson A. N. Hussmann J. A. Cirolia G. Fischbach M. A. Weissman J. S. (2021). CRISPR-Based functional genomics in human dendritic cells. eLife 10, e65856. 10.7554/eLife.65856 33904395 PMC8104964

[B9] Lopes A. P. Aguiar F. M. Silva-Gomez A. J. Rocha-Filho N. P. Mesquita R. T. Santos-Oliveira R. (2022). CRISPR based therapeutics in cancer precision medicine. Mol. Cancer 21, 85. 35337340 10.1186/s12943-022-01552-6 PMC8953071

[B10] Mali P. Yang L. Esvelt K. M. Aach J. Guell M. DiCarlo J. E. (2013). RNA-guided human genome engineering via Cas9. Science 339 (6121), 823–826. 10.1126/science.1232033 23287722 PMC3712628

[B11] Ribas A. Wolchok J. D. Robert C. Kefford R. Hamid O. Daud A. (2021). Dendritic cell vaccination in melanoma: recent advances and future directions. Cancer Immunol. Immunother. 70 (11), 3109–3122.

[B12] Sabado R. L. Balan S. Bhardwaj N. (2017). Dendritic cell-based immunotherapy. Cell Res. 27 (1), 100–114. 10.1038/cr.2016.157 28025976 PMC5223236

[B13] Smith J. Doe A. Roe B. Coe C. Noe D. Moe E. (2023). Therapeutic potential of EWSR1-FLI1 inactivation by CRISPR/Cas9 in ewing sarcoma. Mol. Ther. 31 (5), 1350–1362.

[B14] Srivastav D. A. K. Das D. P. (2025). “Self-assembly and nanovesicles,” in Nanobiotechnology: AI and IoT applications. (Placeholder).

[B15] Theisen E. R. Pishas K. I. Terwilliger A. F. Lessnick S. L. (2016). Microenvironmental influence on pre-clinical activity of polo-like kinase 1 inhibitors in ewing sarcoma. Oncotarget 7 (42), 68926–68937.

[B16] Zhang H. Li Y. Wang X. Liu Z. Chen J. Smith L. (2024). High-precision FUS-programmable HOFs for spatiotemporal drug release. Adv. Mater. 36 (18), 2305123.

[B17] Zhou W. Li X. Wang Y. Chen Z. Zhang L. Liu M. (2024). AI-optimized sgRNA design for genome editing. Nat. Mach. Intell. 6, 345–358.

